# Autosomal Recessive Infantile Hyaline Fibromatosis Identified Using Artificial Intelligence-Assisted Rapid Whole Genome Sequencing: A Rare, Multisystemic, Hereditary Disorder

**DOI:** 10.7759/cureus.62037

**Published:** 2024-06-09

**Authors:** George X Ye, Eric Ontiveros, Axel Ivander, Milen Velinov, Christopher Simotas

**Affiliations:** 1 Pediatrics, Rutgers Cancer Institute of New Jersey, New Brunswick, USA; 2 Pediatrics, Rutgers Robert Wood Johnson Medical School, New Brunswick, USA; 3 Clinical Genomics, Rady Children's Hospital, San Diego, USA; 4 Pediatrics, Robert Wood Johnson University Hospital, New Brunswick, USA

**Keywords:** antxr2 gene, fabric gem®, rapid whole-genome sequencing, pediatric gastroenterology, pediatric rheumatology, rare case report, artificial intelligence, genetics, pediatrics

## Abstract

Infantile hyaline fibromatosis syndrome (HFS) is an ultra-rare genetic condition characterized by the deposition of hyaline material in the skin, muscle, and viscera. Potential complications include debilitating joint contractures, coarse facial features, recurrent infections, failure to thrive, and death. Here, we present the case of a six-month-old infant with a history of painful extremity contractures, global developmental delay, neck hemangioma, and feeding intolerance presenting to our institution with abdominal distension. The multi-systemic, rapidly progressing, severe nature of her symptoms prompted consultation with inpatient pediatric genetics. Per their recommendation, rapid whole-genome sequencing (rWGS) was done with Fabric GEM®-assisted artificial intelligence (Fabric Genomics, Oakland, California, United States) at Rady Children’s Hospital Institute for Genomic Medicine (San Diego, California, United States), revealing homozygous pathogenic variant c.652T>C; P.Cys218Arg in the *ANTXR2* gene consistent with HFS. This case was significant not only for its rarity, but also its early manifestation of symptoms, wide range of affected body systems, and severity of symptoms, which together present a fascinating diagnostic dilemma for future clinicians that should be taken into consideration. It also highlights the increasing utility of AI-assisted rWGS as a diagnostic tool for medically complex patients with unknown multisystemic hereditary conditions.

## Introduction

According to the United States (US) Orphan Drug Act, a rare disease is defined as a condition impacting less than 200,000 people nationwide [1]. However, there is no current consensus for defining an ultra-rare disease. Some researchers propose they be defined as those with a prevalence of less than one in 50,000 (based on 2023 population figures, fewer than 6,640 people in the US) [2]. 

Hyaline fibromatosis syndrome (HFS), also known as inherited systemic hyalinosis or *ANTXR2*-related HFS, meets such criterion for an ultra-rare condition, affecting fewer than one in 1,000,000 births throughout the US and with fewer than 100 total cases reported worldwide [3]. It is inherited in an autosomal recessive manner and characterized by mutations in the *ANTXR2* (Anthrax Toxin Receptor 2) gene, resulting in the abnormal deposition of hyaline material in skin and organs [3]. 

Symptoms can subsequently develop in multiple organ systems including rheumatological (progressive joint contractures), musculoskeletal (severe motor disability), immunological (increased susceptibility to infections), dermatological (thick, rough skin, hyperpigmented macules, nodules, and papules), and gastrointestinal (failure to thrive, nausea, vomiting, protein-losing enteropathy, failure to thrive). Complications from these can result in lifelong debilitation and early death.

## Case presentation

A four-month-old female child was referred to our pediatric ED by the outpatient hematologist-oncologist of our institute for further evaluation of failure to thrive in the setting of abdominal distension and global developmental delay. She was born at an outside hospital at 38w0d gestation via spontaneous vaginal delivery to a G5P5 mother with no significant medical history. The mother and father were both immigrants from the same village in Afghanistan but denied consanguinity. This pregnancy was complicated by gestational diabetes, iron deficiency anemia, and gestational hypertension, and the mother was taking oral vitamin B12 and IV iron at the time of delivery. Delivery was complicated by the nuchal cord but APGAR was 7/9 at birth and initial postpartum course was uncomplicated. New Jersey State Newborn Screen was negative, and the child was discharged home on day 3 of life with routine care instructions. 

**Figure 1:**
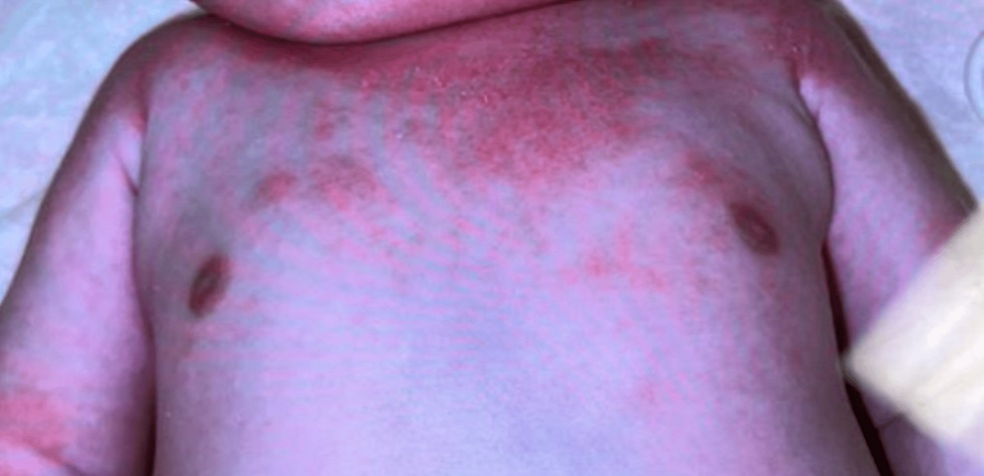
Joint Contractures of Upper Extremities

Starting at two months of age, the child was noted to have abnormal neurological development, including bilateral upper extremity hypotonia, weak palmar grasp, pain with all passive and active extremity movements, and bilateral contractures throughout the hands, feet, arms, and legs. She was unable to extend her limbs fully or tolerate diaper changes without crying (Figure 1).

**Figure 2:**
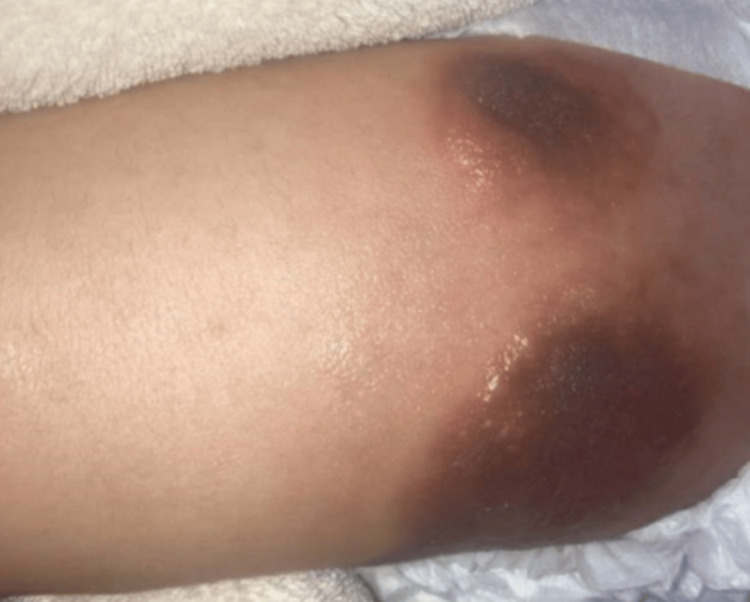
Skin Lesions on Thigh

All four extremities and torso had diffuse areas of blotchy bruising, hyperpigmented macules, and rough eczematous skin (Figure 2).

**Figure 3:**
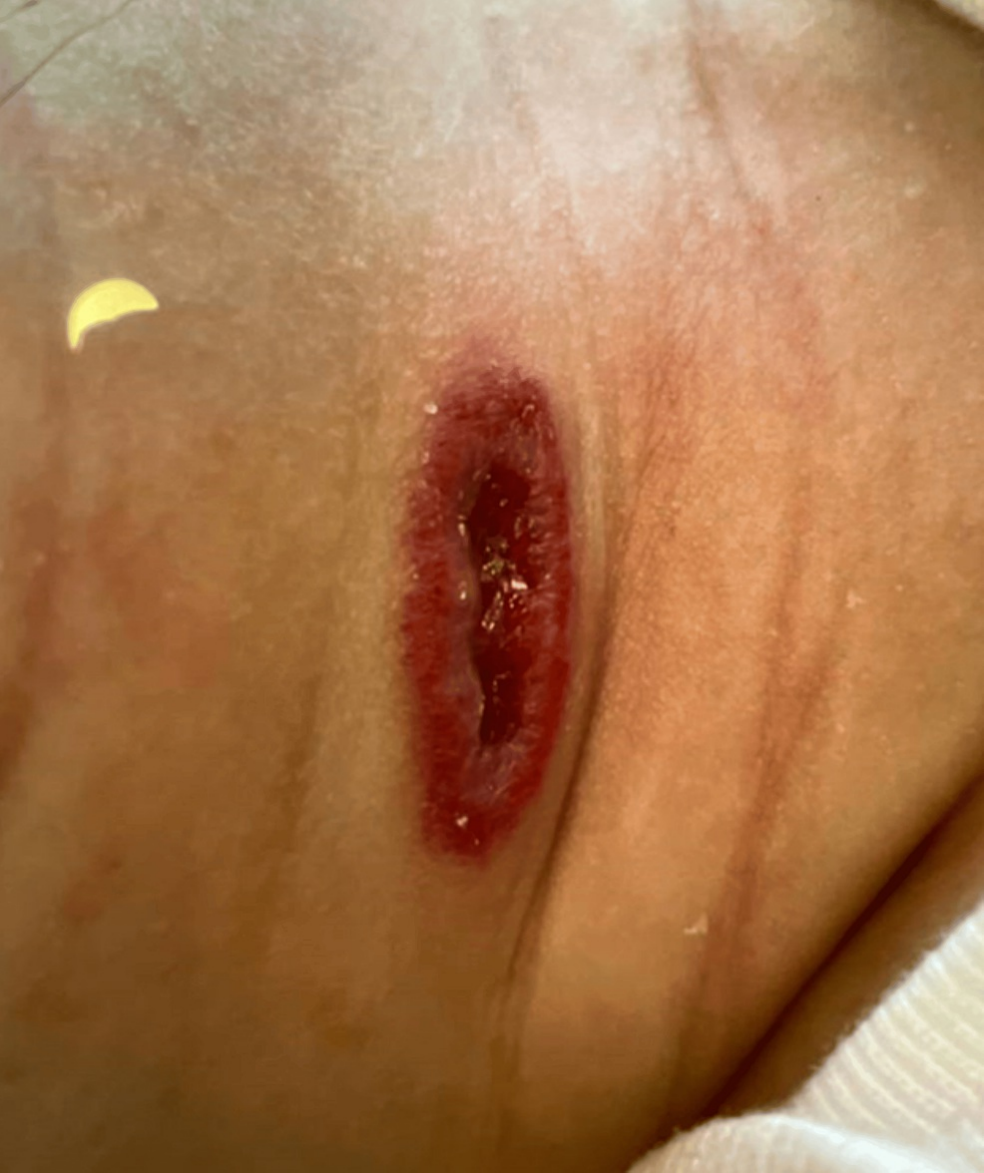
Hemangioma on Neck Prior to Propanolol Use

An outside hospital neurologist was consulted who recommended a head and spine MRI; this showed no significant anomaly and the child began working with Physical Therapy and Occupational Therapy as an outpatient. At three months of age, the child was seen as an outpatient by our institution's hematologist-oncologist for 2x0.8 cm bleeding, ulcerated infantile hemangioma in the center of the neck (Figure 3), for which she was started on propranolol ointment and mupirocin.

At the outpatient Hematology-Oncology follow-up when she was four months old, the child was noted to have several new symptoms including diminishing appetite (now reliant on syringe feeds by mother, declining from 25th to 6th percentile for age), abdominal distension (splenomegaly, hepatomegaly), and worsening of contractures and hypotonia bilaterally. Given these concerns, the hematologist referred the child directly to our ED and she was subsequently admitted as an inpatient for a comprehensive workup including neurology, gastroenterology, and genetics referrals. 

Initial laboratory studies obtained, including complete blood count (CBC), comprehensive metabolic panel (CMP), magnesium, phosphorus, blood culture, lactate dehydrogenase (LDH), coagulation factors, creatine kinase (CK), and uric acid, were significant for elevated WBC 25.5, platelets 853, and D-dimer 6,128. Ultrasound of the abdomen, head, and neck showed no anomalies besides mild ileus and simple hemangioma without fluid collection or mass. Pediatric Neurology, Genetics, and Gastroenterology consults were obtained, with consensus broad working diagnosis categories of primary neurological (prenatal akinesia, arthrogryposis), rheumatologic (chronic infantile neurological cutaneous articular (CINCA) syndrome, neonatal-onset multisystem inflammatory disease (NOMID)), metabolic (congenital glycosylation disorder), or genetic (chromosomal disorder).

Blood work for multiple genetic panels was sent out including carbohydrate-deficient transferrin, prenatal akinesia arthrogryposis panel, and chromosomal microarray. Over the following week, the child was started on nasogastric tube feeds and showed improvement in weight gain with regular, formed bowel movements. Hemangioma size was noted to be stable. The child was subsequently discharged from the inpatient unit to a long-term rehabilitation hospital for feeding support and physical therapy. 

**Figure 4:**
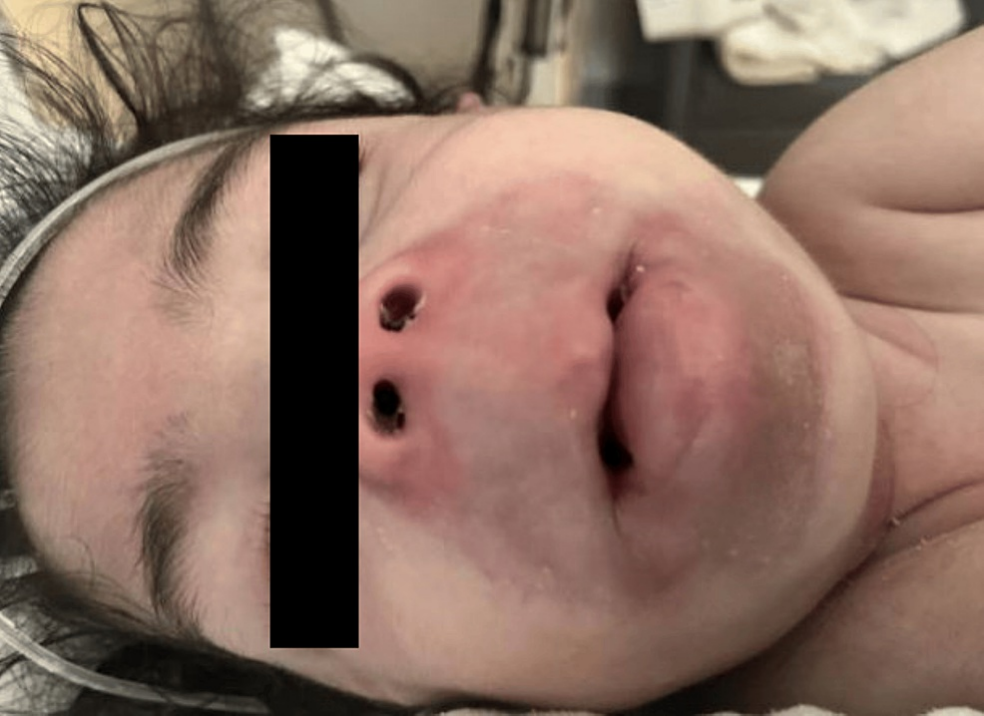
Perioral Hyaline Deposits and Swelling

**Figure 5:**
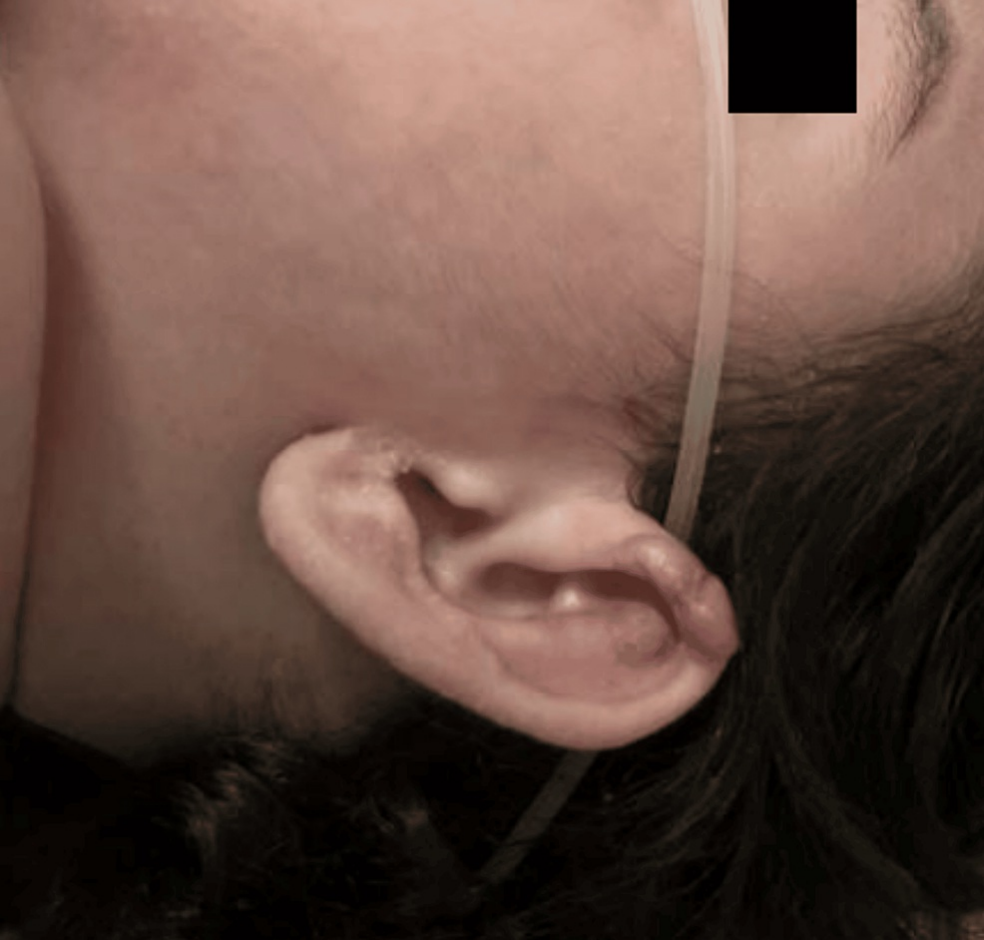
Hyaline Deposits on Left Ear

Per parental request, at four months of age, the patient was seen at an outside hospital for a Rheumatology consultation. Her exam was significant for syndromic appearing facies including bilateral epicanthal folds, torticollis, cheilitis, periorbital swelling and ear growths (Figures 4, 5), gingival inflammation, progressively worsening pain and contractures in the bilateral joints, and generalized hypotonia, prompting admission at this institution for continued workup. The patient was started on gabapentin, Motrin, and Tylenol for pain relief, and whole exome sequencing approval was requested. She was discharged back to the rehabilitation hospital for further care.

Two weeks following this admission, the patient was transferred urgently to our ED for acute onset tachypnea and retractions. She tested positive for adenovirus and was admitted to the inpatient unit for supportive care. During this admission, the mother expressed frustration that the extensive workup at both hospitals had not yielded a unifying diagnosis despite the high suspicion. The child continued to struggle with severe pain in her joint contractures, as well as new onset diarrhea and worsening non-bloody, non-bilious emesis despite the addition of multiple reflux and motility medications.

**Figure 6:**
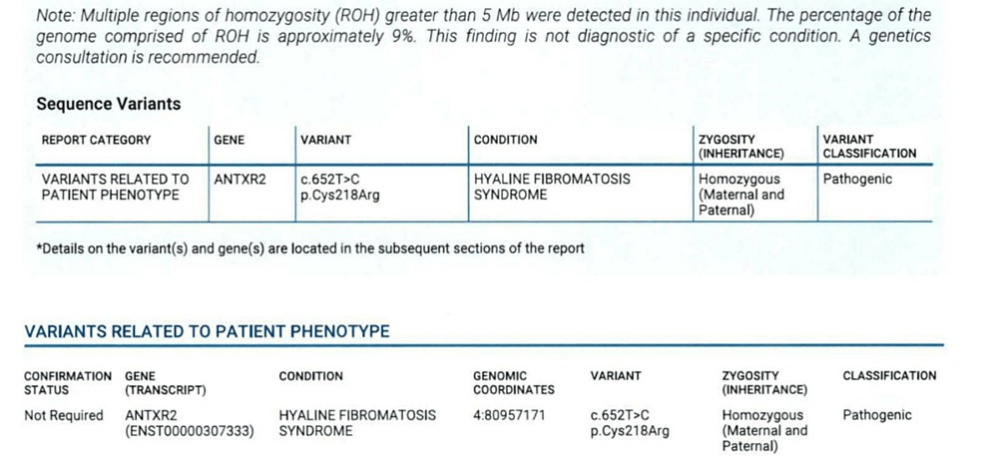
Whole Genome Sequencing Results (excerpt)

At this time, results of initial genomic panels and microarray sent from our institution were still pending and anticipated to take longer than one month to result, while whole exome sequencing at an outside hospital was rejected due to lack of insurance coverage. Therefore, the inpatient genetics team was re-consulted and recommended a request for rapid whole genome sequencing (rWGS) through Rady Children’s Hospital in San Diego, California. The sample was obtained and shipped on Thursday, January 5, 2023; on Tuesday, January 10, 2023, Rady Institute for Genomic Medicine notified our institution of the preliminary results showing a homozygous pathogenic mutation in the *ANTXR2* gene, later specified to be c.652T>C; P.Cys218Arg, consistent with infantile HFS (Figure 6). A multidisciplinary meeting was held the same day with all consulting teams to convey the implications of these findings, coordinate gastrostomy tube placement, and discuss long-term goals. 

## Discussion

Infantile HFS: diagnosis and clinical characteristics

Infantile HFS is an autosomal recessive disorder characterized by abnormal deposition of hyaline deposits throughout the external body and internal organs. Although the exact mechanism of this disease is unknown, one theory suggests mutation in *ANTXR2* results in extravasation of hyaline substances through the damaged basement membrane to the extracellular matrix, thus resulting in the characteristic deposits, joint contractures, and visceral damage. *ANTXR2* is known to encode for a transmembrane protein binding to both laminin and collagen IV, which plays a role in the adhesion of the basement membrane to the extracellular matrix [4]. The resulting complications are multisystemic, debilitating, and progressive in nature (Table 1).

**Table 1:** Phenotypic Features of Autosomal Recessive Infantile Hyaline Fibromatosis

Body System	Symptoms
Dermatological	Thickened, rough skin, hyperpigmented macules, pearly papules of face and neck. Hyaline nodules in joints, muscles, and internal organs
Gastrointestinal	Failure to thrive, nausea, vomiting, protein-losing enteropathy, perianal masses
Rheumatological	Progressive, painful joint contractures
Musculoskeletal	Severe motor disability
Immune	Increased susceptibility to infections
ENT	Coarse facial appearance, depressed nasal bridge, ear malformations (large or low-set ears, preauricular skin tags)
Skeletal	Osteopenia, multiple fractures
Dental	Gingival Hypertrophy

It has been shown that HFS is disproportionately prevalent in Middle Eastern, North African, and Indian communities, especially in those with a high frequency of consanguineous marriages [3]. In this case, although the child’s mother initially denied being related to the father of the child, whole genome single nucleotide polymorphism (SNP) microarray analysis showed extended contiguous regions of allele homozygosity equivalent to a third-degree parental relationship (for example, first cousins). Extensive genetic counseling for parents desiring future children (due to the 25% risk of offspring of carrier parents developing the condition) is thus essential. 

There is a direct link between the age of symptom manifestation and the risk of morbidity and mortality in HFS [3]. While the juvenile form of the disease (diagnosed after the age of seven years) can present with comparatively mild symptoms, the infantile form (diagnosed at birth or soon afterward) results in severe debilitation, multisystem involvement, and frequently early death before the age of two [5,6]. 

Unfortunately, there is no cure for juvenile or infantile HFS, and treatment strategies are largely supportive. The characteristic hyaline deposit complexes often recur following radiotherapy or surgical excision. Attempts to prevent further growth using intralesional or systemic steroids have seen limited success [7-9]. Ultimately, careful coordination of multidisciplinary care is essential for the management of this complex condition, such as assistance from gastroenterology for feeding therapy (with enteral nutrition if necessary), nutritional supplementation to prevent fractures and promote growth, and regular physical and occupational therapy for contractures [8,10]. 

Several diagnostic studies can assist in the diagnosis of HFS. For example, a skin biopsy can show the characteristic accumulation of hyaline deposits in the dermal layer, as well as copious periodic acid-Schiff (PAS)+ staining fibroblasts [6]. Intestinal biopsy often demonstrates atrophy of the villi and lymphangiectasia (resulting in protein-losing enteropathy). Plain-film X-ray of the spine, meanwhile, can reveal periosteal reaction, multiple radiolucent lesions, and generalized osteopenia. However, due to the pathognomonic significance of the *ANTXR2* mutation, molecular genetic analysis remains the ultimate diagnostic tool. 

In the current case, the patient’s first symptoms of joint stiffening*, *pain, contractures, and hypotonia appeared very early at the age of two months. Within six months, she developed multiple new, life-threatening manifestations including oral feeding intolerance, recurrent emesis, diarrhea, failure to thrive, and respiratory failure 2/2 viral infection. The early presentation and rapid progression of symptoms strongly suggest infantile as opposed to juvenile HFS, and thus high likelihood of morbidity and mortality. Thus, it was especially crucial for her condition to be diagnosed as quickly as possible. As infantile HFS is an ultra-rare condition with a complex differential diagnosis, rWGS is a valuable tool to rapidly identify the disease. 

AI-assisted rWGS, a novel and promising technology

In the past 10 years, rWGS has transformed the landscape of molecular genetic analysis. High throughput techniques (also known as massively parallel or next-generation sequencing) use technology such as pyrosequencing or Illumina dye sequencing to obtain accurate results in a fraction of the time of traditional methods. Both techniques use real-time “sequencing by synthesis” rather than endpoint polymerase chain reaction (PCR) analysis as in the traditional chain termination or Sanger sequencing processes but in slightly different ways. Pyrosequencing works by quantifying the light generated during the synthesis of complementary DNA strands by enzymatic conversion of pyrophosphate rather than during chain termination as in Sanger sequencing. Illumina dye sequencing, on the other hand, uses a principle named bridge amplification where multiple groups of DNA “libraries” bind to primers on the flow cell surface and the addition of complementary fluorescent bases is measured during replication. 

Compared to traditional methods such as targeted gene analysis and microarray, rWGS can lead to drastically reduced turnaround time and reduction in number of individual assays needed, while sensitivity is roughly equivalent to that of microarray [11-14]. Despite these dramatic improvements in sequencing speed over the past decade, whole genome sequencing clinical interpretation remains a relatively manual process. A major bottleneck is the number of variants that need to be reviewed per case in terms of the patient phenotype and genotype information [12,15]. 

To alleviate this issue, Rady Children Institute of Genomic Medicine is utilizing the Fabric GEM® artificial intelligence analysis tool (Fabric Genomics, Oakland, California, United States), together with an optimized Phevor (Phenotype Driven Variant Ontological Reranking tool)-based analysis (Fabric Genomics) to identify genomic variation in clinical cases (PMID: 34645491, 24702956). GEM leverages a probands’ genotype and phenotype information and ranks disease-causing variants using the Bayes factor, while Phevor works by combining different ontology databases with the output of variant prioritization tools to rank genes and assign a Phevor score. Together these pipelines allow for expedited genome interpretation [12,14,15].

Variants with a GEM score ≥ 0 were reviewed for this proband leading to the identification of a homozygous c.652T>C (p.Cys218Arg) variant in the *ANTXR2* gene with a score of 4.7. Bayes factor scores greater than 2 are considered strong support and provides guidance to analysts on what variants should be prioritized in the analysis process. This variant was also identified by Phevor and has a Phevor score of 1. Variants with a Phevor score ≥ 5 provide further guidance on what variants should be prioritized in the analysis process. Using a combination of next-generation sequencing and these novel artificial intelligence methods, Rady has been able to lower the total turnaround time for commercial rWGS to less than five days, compared to one to two weeks for much more limited results from chromosomal microarray and four to six weeks for targeted gene panels [11]. 

In the past two years, our hospital system based in central New Jersey has successfully sent multiple patient samples from acutely ill children with high suspicion for genetic or metabolic conditions for analysis at Rady Children Institute of Genomic Medicine, resulting in rapid and successful identification of ultra-rare conditions such as the one described here. 

In summary, this case has numerous implications and is significant not only for its rarity, but also for the early manifestation of symptoms, wide range of affected body systems, and severity of symptoms, which together present a fascinating diagnostic dilemma for future clinicians. It also highlights the increasing utility of rWGS as a diagnostic tool for medically complex patients with unknown multisystemic hereditary conditions. 

## Conclusions

There are several reasons why this case is a significant contribution to the literature on an ultra-rare disease, HFS. These include early manifestation of symptoms, a wide range of affected body systems, and high symptom severity. Together, these present a fascinating diagnostic dilemma for future clinicians that should be taken into consideration. It also highlights the increasing utility of a novel technology, artificial intelligence-assisted rWGS, as a diagnostic tool for medically complex patients with unknown multisystemic hereditary conditions.
